# No increase in small-solute transport in peritoneal dialysis patients treated without hypertonic glucose for fifty-four months

**DOI:** 10.1186/s12882-017-0690-7

**Published:** 2017-08-31

**Authors:** Dominique Pagniez, Alain Duhamel, Eric Boulanger, Celia Lessore de Sainte Foy, Jean-Baptiste Beuscart

**Affiliations:** 1 CHU Lille, Nephrology Department, F-59000 Lille, France; 2 Univ. Lille, EA 2694 - Santé publique: épidémiologie et qualité des soins, F-59000 Lille, France; 3 Univ. Lille, Inserm, CHU Lille, U995 - LIRIC - Lille Inflammation Research International Center, F-59000 Lille, France

**Keywords:** Glucose exposure, Glucose sparing, Small-solute transport, Peritoneal dialysis, Peritoneal equilibration test

## Abstract

**Background:**

Glucose is widely used as an osmotic agent in peritoneal dialysis (PD), but exerts untoward effects on the peritoneum. The potential protective effect of a reduced exposure to hypertonic glucose has never been investigated.

**Methods:**

The cohort of PD patients attending our center which tackled the challenge of a restricted use of hypertonic glucose solutions has been prospectively followed since 1992. Small-solute transport was assessed using an equivalent of the glucose peritoneal equilibration test after 6 months, and then every year. Study was stopped on July 1st, 2008, before use of biocompatible solutions. Repeated measures in patients treated with PD for 54 months were analyzed by using (1) the slopes of the linear regression for D_4_/D_0_ ratios over time computed for each individual, and (2) a linear mixed model.

**Results:**

In the study period, 44 patients were treated for a total of 2376 months, 2058 without hypertonic glucose. There was one episode of peritoneal infection every 18 patient-months. The mean of slopes of the linear regression for D_4_/D_0_ ratios was found to be significantly positive (Student’s test, *p* < .001) and the results of the mixed model reflected a similar significant increase for D_4_/D_0_ ratios over time. These results reflected a significant decrease of small-solute transport.

**Conclusion:**

In this large series, minimizing the use of hypertonic glucose solutions was associated in patients on long term PD with an overall decrease of small-solute transport within 54 months, despite a high rate of peritoneal infection.

**Electronic supplementary material:**

The online version of this article (10.1186/s12882-017-0690-7) contains supplementary material, which is available to authorized users.

## Background

Glucose is widely used as an osmotic agent for peritoneal dialysis (PD) because it is inexpensive and reasonably safe in the short term. Its long-term use, however, is associated with both systemic and peritoneal untoward effects, partly mediated by glucose degradation products (GDP) generated during the manufacturing process for dialysate bags.

Systemic consequences of glucose use include hyperglycemia, hyperinsulinemia, dyslipidemia, oxidative stress and the metabolic syndrome [1]. GDP exposure may accelerate the decline in residual renal function and cause fluid overload [2]. All these processes contribute to develop cardiovascular disease and thus reduce patient survival. Regarding the peritoneal membrane, glucose use is commonly associated with an increased small-solute transport (SST) linked to vascular proliferation, less commonly with a reduced osmotic conductance linked to interstitial fibrosis, and in some cases with the development of encapsulating peritoneal sclerosis [3]. One tends to consider each of these changes as a risk factor for the development of the next and less common one [3].

The concept of glucose sparing in PD [1] was introduced in an attempt to minimize the metabolic consequences of glucose absorption. Obtaining ultrafiltration with icodextrin instead of glucose is beneficial in that matter [1]. Biocompatible glucose-based solutions are of interest as they contain less GDP [4]. Low-glucose dialysis regimens using icodextrin and amino-acid containing solutions in combination with biocompatible glucose solutions have been recently developed [5]. It may be difficult to determine if their effects are related to the use of solutions containing less glucose or to the non-glucose solutions themselves. To our knowledge, no previous study has addressed the effects of a deliberate reduction in exposure to hypertonic glucose on peritoneal membrane.

This article reports the evolution of peritoneal SST in 44 patients treated for more than 54 months at our center which is involved in a process for a restricted use of hypertonic glucose dialysis solutions.

## Methods

### Management of PD patients in our center

PD was first introduced in our center at Calmette Hospital in Lille in 1981. In 1988, following the publication of images illustrating glucose-damaged peritonea [6], we decided to tackle the challenge of minimizing the use of hypertonic glucose solutions in PD patients. Since then, all our patients have indeed started PD using only low-strength (1.36%) glucose solutions. A progressive restriction in dietary salt was advised. A dry night was used when nighttime bags were heavily reabsorbed. Furosemide, up to 500 mg BID, was used routinely to sustain residual renal function. Nephrotoxic drugs, such as aminoglycosides for peritoneal infections or nonsteroidal anti-inflammatory drugs, were systematically avoided. Hypertonic (3.86%) glucose solutions were initiated as late as possible, and systematically stopped at the first sign of peritoneal infection. More than one hypertonic bag per day was never used, and during the first four years of the present study, from 1992 to 1996, patients were transferred to hemodialysis if overhydration persisted. Since its availability in 1997, icodextrin has been started only when necessary to keep fluid balance, hypertonic glucose solutions being kept as a second-line defense against overhydration, particularly in anuric patients. The use of intermediate (2.27%) glucose solutions was rare.

### Peritoneal permeability tests

To assess peritoneal permeability, we initially used an equivalent of the glucose PET test, using 3.86% glucose solution, which was described in 1986 as peritoneal absorption curves [7]. It was not concerned with creatinine, and no emphasis was made on ultrafiltration measurements. We later started measuring creatinine as in the PET test, but kept using 3.86 glucose solution. D/P creatinine results were thus not available in the first years of our study. We then found that the interference of glucose with creatinine measurement [8] varied considerably during the study period, probably due to changes of technique at our laboratory, which made use of these data difficult. We also found it difficult to measure ultrafiltration volume reproductively in the thousand of tests performed on the whole cohort during the study period. For all these reasons, we decided to focus on glucose.

The ratio of peritoneal glucose concentrations at 4-h dwell time to peritoneal glucose concentrations at 0-h dwell time (D_4_/D_0_ ratio) was used as an index of SST. The higher the D_4_/D_0_ ratio, the lower was the SST, illustrating a hypopermeable peritoneal membrane. Peritoneal permeability was investigated 6 months after starting PD, and then on a yearly basis. In case of peritoneal infection or clinical acute problem, the test was delayed for 2 months.

### The Calmette hospital cohort

The Calmette Hospital cohort has been followed prospectively since January 1st, 1992. Demographic characteristics, primary renal diagnosis, comorbidity including diabetic status, and the occurrence of intercurrent events, such as peritoneal infections or PD drop-outs and their causes, were recorded and transmitted to the French Peritoneal Dialysis Registry [9]. Peritoneal infections were defined as the presence of white blood cells (≥100 cells/mL) in the dialysate with clinical symptoms or a positive culture. The occurrence of encapsulating peritoneal sclerosis was also recorded. Additionally, data were collected every 6 months, including plasma albumin and C-reactive protein concentrations, peritoneal and renal clearances of urea and creatinine.

For this current paper, we focused on a follow-up of the Calmette Hospital Cohort until July 1st, 2008. At that time, patients started to use biocompatible solutions (Physioneal®), which created a confounding factor. All incident PD patients attending our center were eligible for this follow-up. Were included all PD patients with at least a 6-month PD treatment (*n* = 304). Exclusion criteria were PD treatment for congestive heart failure without terminal renal insufficiency (*n* = 5), and refusal of regular follow-up (*n* = 6). Thus the Calmette Hospital Cohort included 293 PD patients on regular follow-up within the study period. Follow-up ended in the event of death, transfer to HD or renal transplantation, transfer to another center, recovery of renal function, or at termination of the study.

### Long-term evolution of peritoneal permeability in PD patients

The aim of the present study was to describe the long-term evolution of peritoneal permeability in our cohort.

We screened all PD patients of the Calmette Hospital Cohort treated for at least 54 months. Such patients should have had one initial peritoneal permeability test at 6 months and then 4 yearly tests. The inclusion criteria for this study were: a minimum of 3 tests during the first 54 months of follow-up, the first one being performed between 6 and 12 months after initiating PD treatment and the last one between 48 and 60 months. Additionally, PD patients treated for more than 7 years were entered into a very long-term follow-up of peritoneal permeability.

The peritoneal glucose exposure was calculated for each patient included in the study. One daily exchange with a hypertonic (3.86%) glucose solution, an intermediate (2.27%) glucose solution, or an icodextrin solution at least 15 days per month accounted for a 1-month exposure for the specified solution.

### Ethics statement

The study was approved by the Ethics Committee for Medical Research of the University Hospital of Lille (Commission nationale de l’informatique et des libertés: CNIL-number 701012-GD).

### Data analysis and statistical methods

Quantitative variables were described by mean and standard deviation (SD) in the case of a normal distribution and by median and interquartile ranges (Q1, Q3) otherwise. The normality of distribution was checked by using descriptive analyses and the Shapiro-Wilk’s test. Qualitative variables were described by frequencies and percentages. Unpaired Student’s t-test was used to compare baseline characteristics of patients treated for at least 54 months with those treated less than 54 months.

Longitudinal changes of SST were investigated over the first 54 months of PD treatment using a linear regression of D_4_/D_0_ ratio over time in two steps. First, the slope of the linear regression for the D_4_/D_0_ ratio over time was computed for each patient. The slope coefficient was used as an index of the SST evolution for each patient. The mean of the slopes was compared to zero using a one sample Student’s t-test as the Shapiro-Wilk test didn’t reject the normal distribution hypothesis. Second, an analysis of longitudinal data was performed using a linear mixed model with random coefficients [10]. Linear mixed model is adapted in situations where the number of repeated measurements differs between the subjects, and allows handling of correlations between the repeated measurements. We used random coefficients because the relationship with time was of great interest. In this model, we considered a fixed linear time effect and included a random intercept and a random linear time effect.

The very long-term evolution of SST in patients who were treated with PD for more than 7 years was analyzed in two ways. In the first way, the analysis was performed separately according two periods: before and after 54 months. In each period the distribution of the slope coefficients was compared to a symmetric distribution with a median equal to zero, using an unpaired Wilcoxon test because the Shapiro-Wilk test could not be performed because of a small sample. A linear mixed model was also performed with a linear time effect, a random intercept and a random linear time effect. In the second way, we fitted a linear mixed model on the whole data from inclusion to end of follow-up. In this model, we considered a linear and quadratic time effect as well as a random intercept, a random linear and quadratic time effect.

All statistical calculations were carried out by means of R using the nlme package.

## Results

### Patients characteristics

**Fig. 1:**
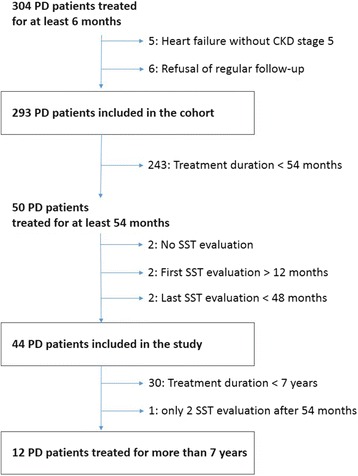
Patient flow

Among the 50 patients treated for at least 54 months, 44 patients fulfilled the criteria for inclusion in our study. None was treated with automated peritoneal dialysis. The patient flow is presented in Fig. 1. Characteristics of included patients are displayed in Table 1 and compared with those of the 243 patients treated less than 54 months. The two groups were similar regarding age, diabetes mellitus, C-reactive protein, diuresis and renal clearances, but serum albumin was significantly higher in included patients (*p* < 0.001). No differences between the two groups were observed for peritoneal characteristics at 6 months (peritoneal clearances and D_4_/D_0_). The frequency of peritoneal infection during the first five years of follow-up was similar in included (1 episode every 18 patient-months) and non included patients (1 episode every 16.2 patient-months). Only one case of encapsulating peritoneal sclerosis occurred in the whole cohort during the study period, and none among included patients. Of the 44 included patients, two (4.5%) had a kidney transplantation, 18 (40.9%) were transferred to hemodialysis, 19 (43.2%) died during the study, and five (10.4%) were still alive on PD as of July 2008, end of the study.

**Table 1:** Characteristics of patients registered in the Calmette Hospital Cohort and included in the study compared with those of non included patients (maintained in peritoneal dialysis less than 54 months)

	Missing data (%)	Included patients (*n* = 44)	Non included patients (PD < 54 months) (*n* = 243)
Age (years)^a^	0	55.8 (13.3)	55.9 (18.8)
Gender (% men)	0	45.5%	57.2%
Diabetes (%)	0	22.7%	27.6%
Albumin (g/L)^a^	18.1	37.4 (3.4)	35.4 (4.7)*
C-reactive protein (mg/L)^b^	5.0	3 [0–10]	3 [0–9.5]
Diuresis (L/24 h)^a^	4.5	1.31 (0.76)	1.31 (0.72)
Renal clearance of urea (mL/min/1,73 m^2^)^b^	5.4	3.7 [2.2–4.7]	3.3 [1.8–5.0]
Renal clearance of creatinin (mL/min/1,73 m^2^)^b^	5.4	7.2 [3.9–9.5]	7.2 [3.7–10.3]
D_4_/D_0_^a^	18.1	0.278 (0.071)	0.288 (0.072)
Peritoneal clearance of urea (L/week)^a^	5.0	46.0 (11.6)	47.6 (11.3)
Peritoneal clearance of creatinin (L/week)^a^	5.0	38.8 (9.4)	38.2 (10.5)

*PD* peritoneal dialysis, *SD* Standard Deviation, *IQR* Interquartile range, *D*_*4*_ */D*_*0*_ ratio of peritoneal glucose concentrations at 4-h dwell time to peritoneal glucose concentrations at 0-h dwell time;

^a^data expressed as mean (SD) – ^b^data expressed as median [IQR]

*P < .001

### Glucose exposure during the first 54 months

**Fig. 2:**
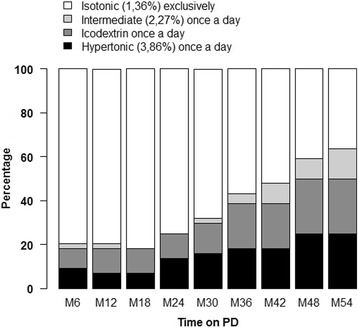
Distribution of included patients according to peritoneal dialysis solutions used in the first 54 months of the study period: hypertonic glucose solution once a day (3.86%) (black), icodextrin solution once a day (dark grey), intermediate glucose solution once a day (2.27%) (light grey), and exclusively isotonic glucose solution (1.36%) (white)

During the 54-month study period, the patients were treated for a total of 2376 months, 2058 months without hypertonic (3.86%) glucose solutions, 2247 months without intermediate (2.27%) glucose solutions, and 2000 months without icodextrin solutions. The association of one hypertonic (3.86%) glucose solution and one icodextrin solution was used in only 3 patients, 2 from the start and 1 from month 42. All in all, these patients were treated only with low-strength (1.36%) glucose solutions for 1703 of the 2376 months. Distribution of patients according to dialysis solutions used during the 54-month study period is shown in Fig. 2. All patients used only conventional glucose solutions (Dianeal ®) during the study period.

### Evolution of D_4_/D_0_ ratio during the first 54 months

**Fig. 3:**
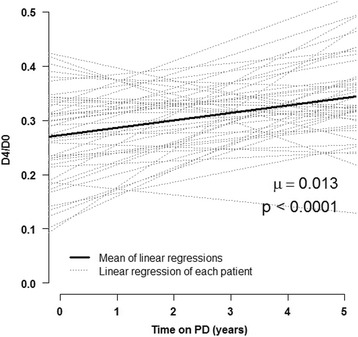
Evolution of peritoneal permeability for 44 patients in the first 54 months of the study period. Light grey lines represent linear regressions of D_4_/D_0_ over time for each patient and black line represents the mean of these linear regressions. μ represents the mean of the slopes

A total of 202 tests were realized in the 44 patients, i.e. an average of 4.6 tests per patient. These data were used to compute the linear regression of D_4_/D_0_ ratio over time for each individual, as described above. The mean of slope coefficients was 0.0136/year 95% CI [0.0069; 0.0202] and was significantly positive (*p* < 0.0001). The coefficient obtained while fitting a linear mixed model with a random intercept and a random time effect was 0.0131/year (95% CI [0.0065; 0.0197]; *p* < 0.0001). These results are displayed in Fig. 3. The D_4_/D_0_ ratio had increased progressively and significantly over time on PD, reflecting a significant decrease of SST during the first 54 months of PD treatment.

### Evolution of D_4_/D0 ratio after 54 months of PD treatment

**Fig. 4:**
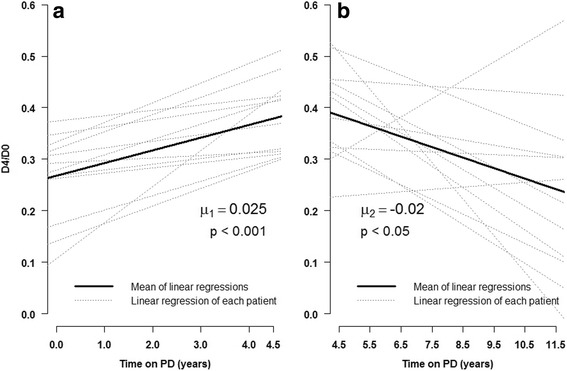
Evolution of peritoneal permeability for the 12 long-term patients in the first 54 months of the study period (part **a**) and beyond this period (part **b**). Light grey lines represent linear regressions of D_4_/D_0_ over time for each patient and black line represents the mean of these linear regressions. μ represents the mean of the slopes

A total of 12 patients were treated with PD for more than 7 years. In these patients, the mean of slope coefficients for the late period was significantly negative (−0.020; 95% CI [−0.038; −0.003]; *p* < 0.05). The coefficient obtained while fitting a linear mixed model with a random intercept and a random time effect was also negative (−0.018; 95% CI [−0.030; −0.005]; *p* < 0.01). This suggested an increase of SST in this late period for these patients. This result is displayed in Fig. 4.

**Fig. 5:**
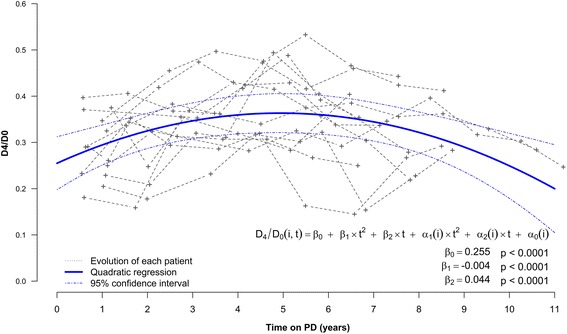
Quadratic regression of D_4_/D_0_ over time (blue bold line). The individual evolution of D_4_/D_0_ over time is represented in grey for each patient

This result was confirmed by the quadratic regression using a linear mixed model applied on all D_4_/D_0_ measures from PD initiation. The quadratic coefficient was significantly negative (−0.004; 95% CI [−0.007; −0.002]; *p* < 0.0001) which confirmed that peritoneal permeability decreased first, and then increased during the late period. Results are presented in Fig. 5 and covariance parameters derived from the model are presented in supplementary Additional file 1: Table S1.

## Discussion

In our cohort of PD patients in whom the use of hypertonic glucose solutions was persistently restricted, we found that, in a subgroup of 44 patients treated for more than 54 months, SST did not increase, but actually decreased, as illustrated by a significant positive mean of slopes for D_4_/D_0_ ratios. Most studies [11–19] have reported a progressive SST increase over time. Known causes of SST increase include peritoneal infection [14] and glucose exposure [19]. Low GDP biocompatible solutions reduce peritoneal inflammation and might prevent deterioration of the peritoneal function [4]. Our findings could not be explained by a low incidence of peritoneal infection, as it was rather high, or by the use of low GDP biocompatible PDFs. At baseline, SST rates in included patients were not different from those in non included patients. We therefore speculate that our findings may be related to the low exposure to hypertonic glucose, i.e. 87% of the first 54-month PD treatment period without resorting to hypertonic peritoneal solutions.

Information on the use of hypertonic glucose solutions is usually not available in longitudinal studies of peritoneal permeability [19]. Davies et al. [20] reported on 22 patients treated 5 years with PD. Among them, 13 patients had stable SST; they had glucose exposure significantly less than the 9 others in whom SST increased. Our findings in a very large number of long-term patients are in accordance with these results. Moreover, they provide evidence that a policy for systematic limitation of the use of hypertonic glucose solutions is possible, and appears to prevent the progressive increase in SST with time in long-term PD patients. One should, however, take into account the fact that patients who stay on PD for a long time may have a better peritoneum, persisting renal function, or lower salt intake. Thus, the extrapolation to the entire population of a potential protective effect of high glucose restriction remains speculative, and should be the object of another study. One may notice, however, that no difference was found in peritoneal characteristics at 6 months between the 44 patients includes in the study and the other patients of the cohort.

The D_4_/D_0_ ratio appears to be an acceptable alternative to D/P creatinine, more convenient for serial monitoring [21]. Additionally, the use of a 3.86% glucose solution for performing the PET has been advocated by the International Society for Peritoneal Dialysis since 2000 [22]. A recent study in 10,142 patients treated mostly with automated PD and followed with the standard PET test showed that D/P creatinine and D_4_/D_0_ ratio were highly correlated, and yielded similar risk prediction. Taking in consideration ultrafiltration volume did not improve risk prediction [23]. In our study, SST initially decreased, more than simply not increasing, in most of our patients.

The late evolution of peritoneal permeability remains of concern. We observed after 54 months a rapid SST increase in the 12 patients with a very long-term follow-up. This may be due to the cumulative effect of glucose exposure, the SST increase becoming the predominant phenomenon*.* The particular severity of late peritoneal infection was also emphasized [24].

Prevention of overhydration was of paramount importance in our patients. Before icodextrin was available, we often resolved to transfer our patients from PD to hemodialysis rather than using hypertonic glucose solution twice a day. Such strategy led to a premature cessation of PD before ultrafiltration loss actually occurred. The availability of icodextrin has facilitated maintenance on PD of anuric patients for extended periods of time.

This study has several strengths. The approach of limiting the use of hypertonic glucose bags was original [25] and applied during a long period. The number of long-term PD patients reported is high. Large longitudinal studies on the time course of peritoneal fluid transport are scarce (18); to our knowledge, none has reported on 44 patients with an average of 4.6 tests each. Regarding the statistical analysis, we used linear mixed models to take into account the clustered nature of the data as each patient observation was a cluster of repeated D_4_/D_0_ measurements.

Obvious limitations of our study are its single-center and observational design without control group, even though no interventional study with control group long enough to compare long-term PD patients has ever been performed. Focusing on long-term patients introduced a selection bias, as previously discussed, and makes the generalization of our conclusions to the average patient hazardous. Adjustment for patients characteristics was not possible, due to the limited number of patients studied. We generally tried to limit the impact of the modification of practices with time, by ending the study before biocompatible solutions were introduced, and by resorting to the use of the D_4_/D_0_ ratio. The beneficial effect of the introduction of icodextrin has already been discussed. Not using D/P creatinine, however, makes direct comparison with other studies difficult.

The present study suggests that restricted use of hypertonic glucose solutions delays the occurrence of SST increase beyond 54 months of PD treatment. Our study ended before the use of low-GDP biocompatible solutions was started. Thus, it is entirely possible that systematic use of low-GDP biocompatible solutions will further delay this occurrence, even if their use is associated with a SST increase in the short term [3, 26]. Anyway, the impact of hypertonic glucose solutions appears sufficient to make it mandatory to stratify studies comparing peritoneal effects of biocompatible solutions with standard solutions according to the number of daily exchanges using hypertonic glucose [26].

## Conclusions

In conclusion, we showed that a persistent restricted use of hypertonic glucose solutions was feasible, and in patients on long term PD was associated with an overall decrease of SST over a 54-month period in spite of a high rate of peritoneal infection. The subsequent evolution of peritoneal permeability remained of concern. Cost/benefit studies assessing the effect of introducing biocompatible solutions at some time in the course of PD treatment would be of interest. Such studies, comparing conventional and biocompatible PD solutions, should be stratified according to the use of hypertonic glucose solutions.

## Additional file

Additional file 1: Table S1. Covariance parameters derived from the linear mixed model fitted on the whole data from inclusion to end of follow-up (PDF 180 kb)

## Abbreviations

95% CI: 95% confidence interval
GDP: Glucose degradation products
PD: Peritoneal dialysis
PET: Peritoneal equilibration test
SD: Standard deviation
SST: Small-solute transport
