# Supplementary material for: No increase in small-solute transport in peritoneal dialysis patients treated without hypertonic glucose for fifty-four months
Source: BMC Nephrol. 2017 Aug 31;18:278. doi: 10.1186/s12882-017-0690-7 (PMC5580320; doi:10.1186/s12882-017-0690-7)
Supplement: Additional file 1: Table S1. — Covariance parameters derived from the linear mixed model fitted on the whole data from inclusion to end of follow-up (PDF 180 kb) [file 12882_2017_690_MOESM1_ESM.pdf]

Supplementary Data

BMC Nephrology

Article: No increase in small-solute transport in peritoneal dialysis patients treated without hypertonic glucose for fifty-four months.

Dominique Pagniez, Alain Duhamel, Eric Boulanger, Celia Lessore de Sainte Foy, Jean-Baptiste Beuscart

S-Table 1: Covariance parameters derived from the linear mixed model fitted on the whole data from inclusion to end of follow-up. In this model, we considered a linear and quadratic time effect as well as a random intercept, a random linear and quadratic time effect.

	Intercept	Time	$(Time)^2$
Intercept	0.00616269	-0.00157265	0.00014259
Time		0.00100794	-9.58505E-05
$(Time)^2$			9.41376E-06
